# Freshwater fishes (Actinopterygii) of Kenyir Reservoir, Peninsular Malaysia: Updated checklist, taxonomic concerns and alien species

**DOI:** 10.3897/BDJ.11.e100337

**Published:** 2023-07-03

**Authors:** Mohamad Aqmal-Naser, Norsyafira Anis Ali, Nur Ummiliani Azmi, Muhammad Fahmi-Ahmad, Syed Ahmad Rizal, Amirrudin B. Ahmad

**Affiliations:** 1 Terrestrial Ecology, Biodiversity and Aquatic Research (TEBAR), Institute of Tropical Biodiversity and Sustainable Management, Universiti Malaysia Terengganu, 21030, Kuala Nerus, Malaysia Terrestrial Ecology, Biodiversity and Aquatic Research (TEBAR), Institute of Tropical Biodiversity and Sustainable Management, Universiti Malaysia Terengganu, 21030 Kuala Nerus Malaysia; 2 Biodiversity and Ecology Research (BERes), Universiti Malaysia Terengganu, 21030, Kuala Nerus, Malaysia Biodiversity and Ecology Research (BERes), Universiti Malaysia Terengganu, 21030 Kuala Nerus Malaysia

**Keywords:** biodiversity, conservation, impoundment, native species, Southeast Asia

## Abstract

**Background:**

A total of 87 freshwater fish species from 30 families were recorded from the Kenyir Reservoir, Peninsular Malaysia, where 75 are native and 12 are introduced species. Few species still have unstable taxonomy identities which urge further studies. Most of the species were categorised as Least Concern (LC) and two were threatened species; Endangered and Critically Endangered (EN and CR). One introduced species, *Gambusiaaffinis* is widespread in the human-associated area, while other introduced fish species can be considered low in numbers.

**New information:**

Twenty five fish species are recorded for the first time in the Kenyir Reservoir.

## Introduction

Reservoirs have generated high economic impact via inland fisheries, especially in the Asian region (Tessier et al. 2016). The number of reservoirs is also expected to increase due to economic development, climate change and human population (Zarfl et al. 2014). This has led to the fragmentation of the major rivers worldwide because of dam construction (Santos et al. 2013). The concern arises whether this fragmentation will be affecting the aquatic ecosystems at temporal and permanent spatial isolation.

Peninsular Malaysia has more than 70 reservoirs used for hydropower and agriculture purposes (Syuhada et al. 2018). One of them is Kenyir Reservoir, formed by damming the Terengganu River from 1978 until its maximum water capacity in 1985. Cramphorn (1983) had studied fish community of the Terengganu River, before the dam was wholly inundated in 1985. Since then, several researchers have conducted several studies to document the fish species recorded in this reservoir (post-inundation) (Ambak and Jalal 1998, Amirrudin et al. 2002, Kamaruddin et al. 2011). As the taxonomic revision and studies on fish advanced, many scientific names have been synonymised which need the latest update and additional information. Apart from that, some species are rarely been recorded and sometimes recorded only once or twice, for the last 40 years.

In this article, we gathered the previously-published data and our current data to review and improve the information on the fish species richness in the Kenyir Reservoir, coupled with some findings on the taxonomic ambiguity, alien invasive species (AIS) and some new records of the fish fauna. This study is essential and aligns with Malaysian Sustainable Development Goal 15 (SDG) which aims to conserve and restore the terrestrial and freshwater ecosystem, as well as to prevent invasive alien species in the water ecosystems.

## Materials and methods

### Study area

**Figure 1.**
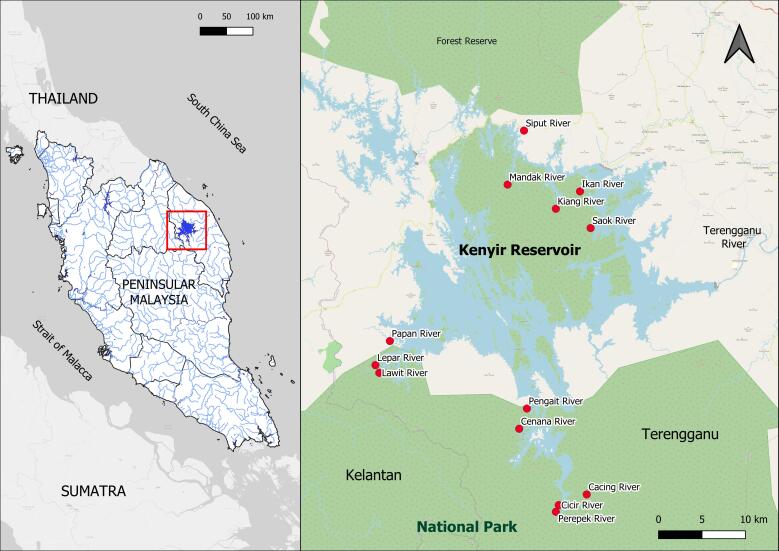
The location of Kenyir Reservoir on the eastern part of Peninsular Malaysia within the red square (left). Streams and rivers sampled haphazardly in 2008, 2013, 2017, 2018, 2019 and 2020 are represented by the red circles (primary data).

Kenyir is the largest freshwater reservoir in Peninsular Malaysia with approximately 260,000 hectares of catchment area (Ambak and Jalal 1998), located on the east coast of Peninsular Malaysia (5°05'20.1"N 102°43'26.5"E) in the Terengganu State (Fig. 1). There were numerous headwater streams with rocky and sandy habitats and more than ten large rivers flowing into Kenyir Reservoir (Ambak and Jalal 2006). The Reservoir comprises of 52.83% forest cover and the other 34.23% is a multitype land-use area (Kamarudin et al. 2018). It experiences northeast monsoon from November until February annually.

### Historical data

The previous checklists on fishes in Kenyir Reservoir were reviewed and all the synonyms and misidentifications were corrected following Zakaria-Ismail et al. (2019) and Fricke et al. (2021). The list includes information on fish reported by Cramphorn (1983), the Department of Fisheries (1994), the Department of Fisheries (1995), Yusoff et al. (1995), Zakaria et al. (1997), Ambak and Jalal (1998), Amirrudin et al. (2002), Shahid et al. (2010) and Kamaruddin et al. (2011).

### Sampling

All sampling events were carried out with the permission by the Department of Fisheries, Terengganu. An electrofishing technique using backpack electro-shocker model LR-24 (Smith Roots Inc.) was used following Aqmal-Naser et al. (in press). The ethical procedure for fish collection follows guideline by Bennett et al. (2016). The data including unpublished data are owned by the corresponding author, collected in 2008, 2013, 2017, 2018, 2019 and 2020. Three units of gill nets (mesh sizes of 1.0, 2.0 and 4.0 inches; 2.5, 5.1 and 10.2 centimeters) were used to collect pelagic fish species and scoop nets were also used to collect small fish-like loaches. Interviews with the boat operators and local fishermen have also been conducted regarding the species that can be found in Kenyir Reservoir (personal communication).

### Species identification

The fish were identified *in situ* when possible based on Zakaria-Ismail et al. (2019). The voucher specimens were fixed in 10% formalin and left for two weeks before rinsing and transferred into 70% alcohol for long-term storage. The preserved specimens were kept in Universiti Malaysia Terengganu Zoological Collection (UMTZC). Species validity and the spelling of the scientific names follow the California Academy of Science’s Catalogue of Fishes (Fricke et al. 2021).

## Checklists

### List of freshwater fish from Kenyir Reservoir

#### Scleropages formosus

(Müller and Schlegel, 1840)

F0C2BEBC-B289-594D-8C03-A381BF549030

##### Materials

- **Type status:** Other material. **Event:** samplingProtocol: Literature; Cramphorn. J. (1983). Department of Fisheries (1994; 1995), Ambak. M.A., Jalal. K.C.A. (1998)

##### Native status

Native species.

##### Conservation status

EN

#### Chitala cf. lopis

(Bleeker, 1851)

F5B2D655-3B6A-59F7-BF0D-60BB9B1F2005

##### Materials

- **Type status:** Other material. **Location:** locality: Cacing River; **Event:** samplingProtocol: Gill net (specimen was not taken), Literature; Cramphorn. J. (1983). Department of Fisheries (1994), Ambak. M.A., Jalal. K.C.A. (1998); year: 2017

##### Native status

Native species

##### Conservation status

NE

##### Notes

**Figure 2.**
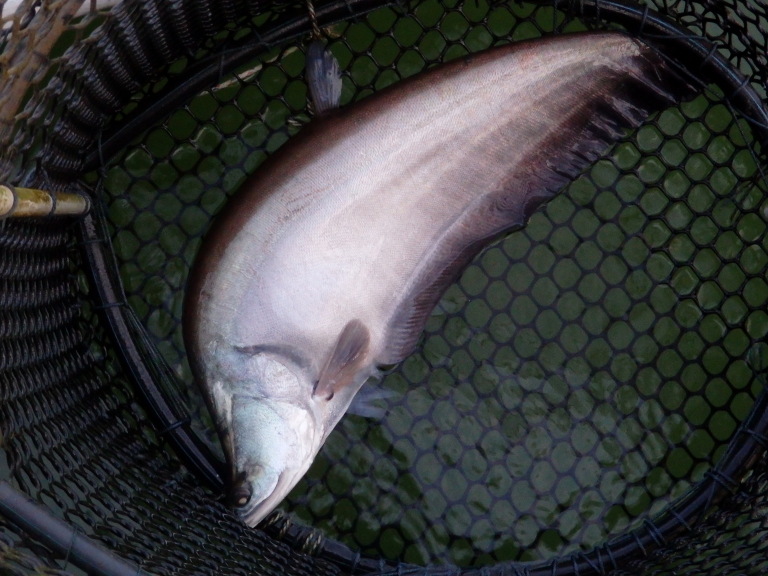
Chitalacf.lopis from Kenyir Reservoir.

The distribution of *Chitalalopis* is restricted to Java, Indonesia (Kottelat and Widjanarti 2005), which raises questions about the current taxonomic identity of other identified *Chitalalopis* outside its native distribution ranges. The species is declared extinct in Java (Ng 2020) as it was not recorded for the past 100 years. The individuals resembling *Chitalaborneensis* were also recorded from Peninsular Malaysia in the Endau and Terengganu River (Mohd Ilham Norhakim Lokman, pers. comm.). The identity of *Chitala* spp. from Peninsular Malaysia needs further taxonomic clarification based on morphology and molecular works (Fig. 2).

#### Notopterus notopterus

(Pallas, 1769)

43F81109-CF3E-51ED-9B81-9ADF80C8545A

##### Materials

- **Type status:** Other material. **Occurrence:** catalogNumber: UMTZC7738; **Location:** locality: Cacing River, Mandak River; **Event:** samplingProtocol: Electrofishing, Literature; Department of Fisheries (1995), Amirrudin. A., Siti Azizah. M.N., Yusri. Y., Norainy. M.H., Mohd Asnizam. A., Ali. A.B. (2002); year: 2017, 2019, 2020

##### Native status

Native species.

##### Conservation status

LC

#### Clupeichthys sp.

10B9F22D-5F8C-5B72-B47C-47B5B634EBEC

##### Materials

- **Type status:** Other material. **Occurrence:** catalogNumber: UMTZC7849; **Location:** locality: Cacing River, Lepar River; **Event:** samplingProtocol: Literature; Department of Fisheries (1994), Aqmal-Naser et al. (2021), Scoop net; year: 2017, 2019, 2020

##### Native status

Native species

##### Conservation status

NE

##### Notes

**Figure 3.**
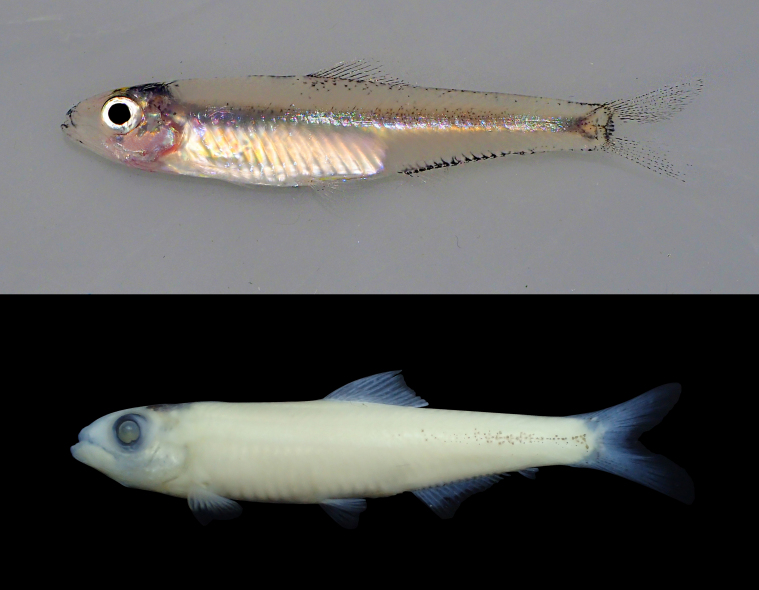
*Clupeichthys* sp. fresh specimen (top) and preserved specimen (bottom).

*Clupeichthysaesarnensis* is restricted to the river systems in Cambodia, Laos, Thailand and Vietnam (Di Dario 2018). The distribution of freshwater clupeid in Peninsular Malaysia, *Clupeichthysperakensis* is restricted to the Perak River system (Ng et al. 2019). Hence, the species recorded in this study (identified as *Clupeichthys* sp.) (Fig. 3) are neither of these species and need further study on their taxonomic identities (Aqmal-Naser et al. 2021).

#### Acantopsis dialuzona

van Hasselt, 1823

079A952F-6C30-592B-880E-8596B27D500C

##### Materials

- **Type status:** Other material. **Occurrence:** catalogNumber: UMTZC7633; **Location:** locality: Cacing River, Lepar River, Lawit River, Siput River, Saok River, Ikan River, Cicir River; **Event:** samplingProtocol: Electrofishing, Literature; Zakaria. M.Z., Yaacob. K.K.K., Noor. J.M. (1997), Ambak. M.A., Jalal. K.C.A. (1998); year: 2017, 2018, 2019, 2021

##### Native status

Native species.

##### Conservation status

LC

#### Acanthopsoides molobrion

Siebert, 1991

416F3D33-458E-5CB1-AE4A-CC00E20F9194

##### Materials

- **Type status:** Other material. **Occurrence:** catalogNumber: UMTZC7676; **Location:** locality: Kiang River, Mandak River, Cicir River, Perepek River; **Event:** samplingProtocol: Electrofishing; year: 2017, 2019

##### Native status

Native species.

##### Conservation status

LC

##### Notes

**Figure 4.**
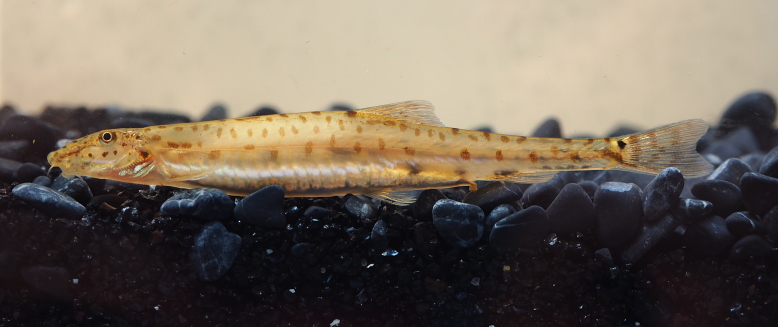
*Acanthopsoidesmolobrion*.

New record to Kenyir Reservoir (Fig. 4).

#### Pangio doriae

(Perugia, 1892)

584CE059-B418-561D-91C3-2F9B50EA7D5F

##### Materials

- **Type status:** Other material. **Occurrence:** catalogNumber: UMTZC7974; **Location:** locality: Perepek River; **Event:** samplingProtocol: Scoop net; year: 2017, 2019, 2020

##### Native status

Native species.

##### Conservation status

LC

##### Notes

**Figure 5.**
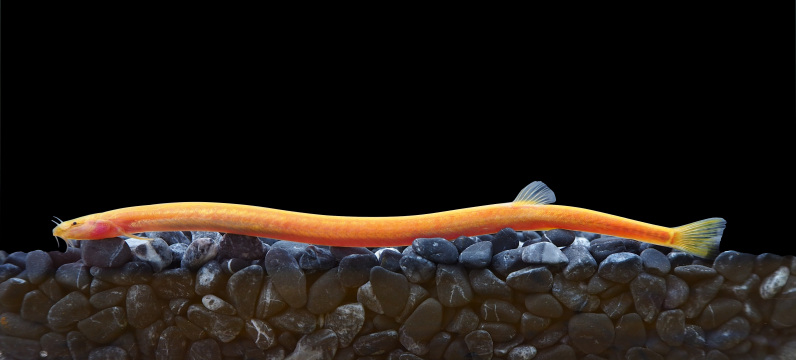
*Pangiodoriae*.

New record to Kenyir Reservoir (Fig. 5).

#### Pangio filinaris

Kottelat and Lim, 1993

213508CB-5B96-5FF5-B9F5-A7FDBEE5D1EF

##### Materials

- **Type status:** Other material. **Occurrence:** catalogNumber: UMTZC7011; **Location:** locality: Siput River, Kiang River, Lawit River; **Event:** samplingProtocol: Electrofishing, Literature; Amirrudin. A., Siti Azizah. M.N., Yusri. Y., Norainy. M.H., Mohd Asnizam. A., Ali. A.B. (2002); year: 2017, 2019

##### Native status

Native species.

##### Conservation status

LC

#### Pangio semicincta

(Fraser-Brunner, 1940)

EF701871-59CB-5CC2-810B-D8D5E0DD7645

##### Materials

- **Type status:** Other material. **Occurrence:** recordedBy: Ambak. M.A., Jalal. K.C.A. (1998); **Event:** samplingProtocol: Literature

##### Native status

Native species.

##### Conservation status

LC

#### Balitoropsis zollingeri

(Bleeker, 1853)

36D68A7C-D450-513B-A28A-E43B2B43C35C

##### Materials

- **Type status:** Other material. **Occurrence:** catalogNumber: UMTZC7689; **Location:** locality: Ikan River; **Event:** samplingProtocol: Electrofishing; year: 2017, 2018, 2019

##### Native status

Native species.

##### Conservation status

LC

##### Notes

**Figure 6.**
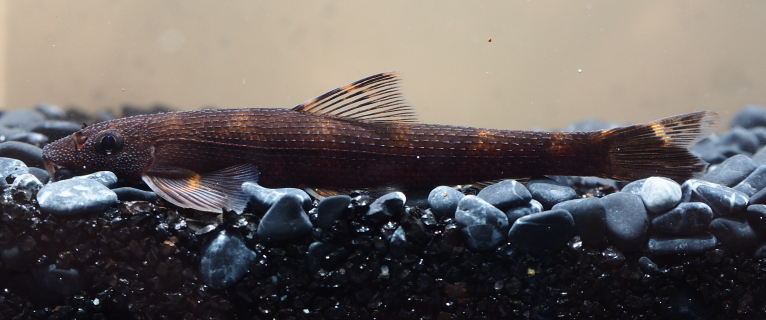
*Balitoropsiszollingeri*.

New record to Kenyir Reservoir (Fig. 6).

#### Homaloptera ogilviei

Alfred, 1967

A0ECD2FD-8234-5E1E-B9C8-3C2BACC8ED02

##### Materials

- **Type status:** Other material. **Occurrence:** catalogNumber: UMTZC7641; **Location:** locality: Siput River; **Event:** samplingProtocol: Electrofishing; year: 2019

##### Native status

Native species.

##### Conservation status

LC

##### Notes

**Figure 7.**
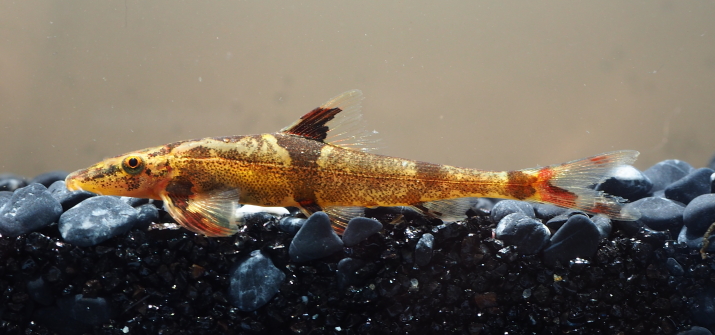
*Homalopteraogilviei*.

New record to Kenyir Reservoir (Fig. 7).

#### Homalopteroides tweediei

(Herre, 1940)

3721274F-1C02-526A-9707-9B8C1CC43495

##### Materials

- **Type status:** Other material. **Event:** samplingProtocol: Literature; Amirrudin. A., Siti Azizah. M.N., Yusri. Y., Norainy. M.H., Mohd Asnizam. A., Ali. A.B. (2002)

##### Native status

Native species.

##### Conservation status

LC

#### Pseudohomaloptera leonardi

(Hora, 1941)

22CA02C9-2C15-5519-BF6A-FB3CEEBB5581

##### Materials

- **Type status:** Other material. **Occurrence:** catalogNumber: UMTZC8681; **Location:** locality: Buluh Nipis River; **Event:** samplingProtocol: Electrofishing; year: 2020

##### Native status

Native species.

##### Conservation status

LC

##### Notes

**Figure 8.**
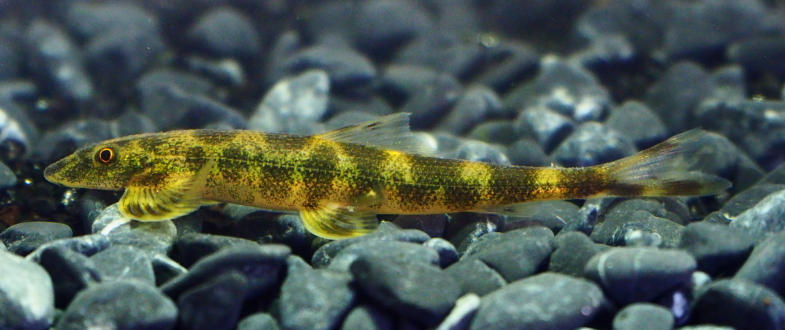
*Pseudohomalopteraleonardi*.

New record to Kenyir Reservoir (Fig. 8).

#### Nemacheilus masyae

Smith, 1933

88073B64-4655-5EAE-9F49-43017F6B948E

##### Materials

- **Type status:** Other material. **Occurrence:** catalogNumber: UMTZC7681; **Location:** locality: Cacing River, Lepar River, Lawit River, Siput River, Kiang River, Cicir River, Perepek River, Cenana River; **Event:** samplingProtocol: Electrofishing, Literature; Cramphorn. J. (1983), Amirrudin. A., Siti Azizah. M.N., Yusri. Y., Norainy. M.H., Mohd Asnizam. A., Ali. A.B. (2002); year: 2017, 2019, 2020

##### Native status

Native species.

##### Conservation status

LC

#### Barbichthys laevis

(Valenciennes, 1842)

1A0CD65D-9AD9-574B-BB06-D059A87EB59A

##### Materials

- **Type status:** Other material. **Event:** samplingProtocol: Literature; Yusoff. F.M., Zaidi. M.Z., Ambak. M.A. (1995), Zakaria. M.Z., Yaacob. K.K.K., Noor. J.M. (1997)

##### Native status

Native species.

##### Conservation status

LC

#### Barbodes rhombeus

(Kottelat, 2000)

48876185-B3E0-5577-BFFD-6305AB5C3F77

##### Materials

- **Type status:** Other material. **Occurrence:** catalogNumber: UMTZC7646; **Location:** locality: Siput River; **Event:** samplingProtocol: Literature; Cramphorn. J. (1983), Ambak. M.A., Jalal. K.C.A. (1998); year: 2017

##### Native status

Native species.

##### Conservation status

LC

#### Barbodes sellifer

Kottelat and Lim, 2021

EE3EA8F9-7176-5278-9AE7-F12504DE1963

##### Materials

- **Type status:** Other material. **Occurrence:** catalogNumber: UMTZC7691; **Location:** locality: Siput River; **Event:** samplingProtocol: Electrofishing; year: 2017, 2018, 2019

##### Native status

Native species.

##### Conservation status

LC

#### Barbodes lateristriga

(Valenciennes, 1842)

9B2A8D00-7C78-59A9-B109-855C3D41D5FA

##### Materials

- **Type status:** Other material. **Event:** samplingProtocol: Literature; Cramphorn. J. (1983), Ambak. M.A., Jalal. K.C.A (1998).

##### Native status

Native species.

##### Conservation status

LC

#### Barbonymus gonionotus

(Bleeker, 1849)

A6FA4051-B502-5945-AC2C-92DD35238A75

##### Materials

- **Type status:** Other material. **Event:** samplingProtocol: Literature; Yusoff. F.M., Zaidi. M.Z. & Ambak. M.A. (1995)

##### Native status

Introduced species. The species was introduced into Kenyir Reservoir through the restocking programme in 1988-1990 by the Department of Fisheries.

#### Barbonymus schwanefeldii

(Bleeker, 1854)

6F3CE8B3-F21A-5D78-8D70-118BB095FB0A

##### Materials

- **Type status:** Other material. **Occurrence:** catalogNumber: UMTZC7661; **Location:** locality: Lawit River, Perepek River, Lepar River; **Event:** samplingProtocol: Electrofishing, Literature; Cramphorn. J. (1983), Ambak. M.A., Jalal. K.C.A. (1998); year: 2017, 2019, 2020

##### Native status

Native species.

##### Conservation status

LC

#### Crossocheilus oblongus

Kuhl and van Hasselt, 1823

C9BE81AD-4F1B-522F-9E4B-2224896F9C21

##### Materials

- **Type status:** Other material. **Occurrence:** catalogNumber: UMTZC7682; **Location:** locality: Perepek River, Ikan River, Kiang River, Siput River, Cenana River, Pengait River; **Event:** samplingProtocol: Electrofishing, Literature; Cramphorn. J. (1983) Department of Fisheries (1994), Yusoff. F.M., Zaidi. MZ., Ambak. M.A. (1995); year: 2017, 2018, 2019

##### Native status

Native species.

##### Conservation status

LC

#### Cyclocheilichthys apogon

(Valenciennes, 1842)

8FE6D2C0-63ED-583B-943E-A98363D03547

##### Materials

- **Type status:** Other material. **Occurrence:** catalogNumber: UMTZC7674; **Location:** locality: Kiang River, Lawit River, Mandak River, Cicir River; **Event:** samplingProtocol: Electrofishing, Literature; Department of Fisheries (1994), Kamaruddin. I.S., Mustafa-Kamal. A.S., Christianus. A., Daud. A., Yu-Abit. L. (2011); year: 2017, 2019, 2020

##### Native status

Native species.

##### Conservation status

LC

#### Cyclocheilichthys armatus

(Valenciennes, 1842)

0C69AA98-FE61-5360-ADA8-23D3F72B9228

##### Materials

- **Type status:** Other material. **Occurrence:** catalogNumber: UMTZC8678; **Location:** locality: Cacing River, Cicir River; **Event:** samplingProtocol: Electrofishing, Gills net; year: 2019, 2018, 2020

##### Native status

Native species.

##### Conservation status

LC

##### Notes

**Figure 9.**
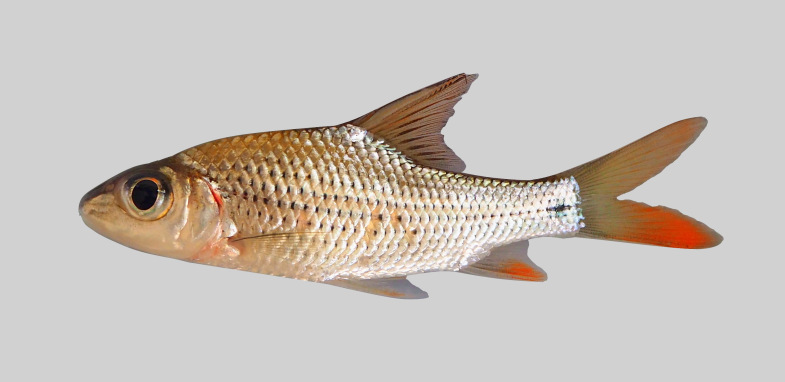
*Cyclocheilichthysarmatus*.

New record to Kenyir Reservoir (Fig. 9).

#### Cyprinus carpio

Linnaeus, 1758

74D35F41-E33A-5F41-8ED5-FC6006F327C0

##### Materials

- **Type status:** Other material. **Event:** samplingProtocol: Literature; Yusoff. F.M., Zaidi. M.Z. & Ambak. M.A. (1995)

##### Native status

Introduced species. The species was introduced into Kenyir Reservoir through the restocking programme in 1988-1990 by the Department of Fisheries.

#### Ceratogarra cambodgiensis

(Tirant, 1884)

FA6E5A4A-6F04-5368-A529-E98FD72D3A4F

##### Materials

- **Type status:** Other material. **Occurrence:** catalogNumber: UMTZC7706; **Location:** locality: Saok River, Ikan River, Siput River; **Event:** samplingProtocol: Electrofishing, Literature; Yusoff. F.M., Zaidi. M.Z., Ambak. M.A. (1995); year: 2017, 2018, 2019, 2020

##### Native status

Native species.

##### Conservation status

LC

#### Hampala macrolepidota

Kuhl and van Hasselt, 1823

ED27AF9A-D4AD-5813-8E89-685162B7A687

##### Materials

- **Type status:** Other material. **Occurrence:** catalogNumber: UMTZC7844; **Location:** locality: Saok River, Cicir River, Siput River, Cenana River, Pengait River; **Event:** samplingProtocol: Electrofishing, Literature; Cramphorn. J. (1983). Department of Fisheries (1995), Zakaria. M.Z., Yaacob. K.K.K., Noor. J.M. (1997); year: 2017, 2019, 2020

##### Native status

Native species.

##### Conservation status

LC

#### Labiobarbus leptocheilus

(Valenciennes, 1842)

B2A91007-1EBD-5DA5-BB76-058E0F7734C9

##### Materials

- **Type status:** Other material. **Occurrence:** catalogNumber: UMTZC7638; **Location:** locality: Lepar River, Kiang River, Mandak River, Siput River, Lawit River, Cicir River, Perepek River; **Event:** samplingProtocol: Electrofishing, Literature; Ambak. M. A., Jalal. K. C. A. (1998), Kamaruddin. I.S., Mustafa Kamal. A.S., Christianus. A., Daud. A., Yu Abit. L. (2011); year: 2017, 2018, 2019, 2020

##### Native status

Native species.

##### Conservation status

LC

#### Lobocheilos rhabdoura

(Fowler, 1934)

3C68E025-F94D-595B-91EC-DCF2AF51BC23

##### Materials

- **Type status:** Other material. **Occurrence:** catalogNumber: UMTZC7766; **Location:** locality: Cacing River, Saok River, Perepek River; **Event:** samplingProtocol: Electrofishing, Literature; Cramphorn. J. (1983), Yusoff. F. M., Zaidi. M. Z., Ambak. M. A. (1995)

##### Native status

Native species.

##### Conservation status

LC

#### Mystacoleucus chilopterus

Fowler, 1935

5B085259-C5A0-5CC5-A5BD-AF1800A7473D

##### Materials

- **Type status:** Other material. **Event:** samplingProtocol: Literature; Cramphorn. J. (1983)

##### Native status

Native species.

##### Conservation status

LC

#### Mystacoleucus obtusirostris

(Valenciennes, 1842)

BBA3E372-E84B-5EDB-91D8-79C46A0B0ECA

##### Materials

- **Type status:** Other material. **Occurrence:** catalogNumber: UMTZC7778; **Location:** locality: Siput River, Kiang River, Lepar River, Saok River, Lawit River, Cicir River, Perepek River, Mandak River; **Event:** samplingProtocol: Electrofishing, Literature; Cramphorn. J. (1983), Ambak. M.A., Jalal. K.C.A. (1998); year: 2017, 2018, 2019, 2020

##### Native status

Native species.

##### Conservation status

LC

#### Neolissochilus soroides

(Duncker, 1904)

50EB25D8-B24C-5F4F-9ADA-18C7AB1046A2

##### Materials

- **Type status:** Other material. **Occurrence:** catalogNumber: UMTZC8679; **Location:** locality: Saok River, Siput River, Pengait River, Cenana River; **Event:** samplingProtocol: Electrofishing, Literature; Yusoff. F.M., Zaidi. M.Z., Ambak. M.A. (1995), Amirrudin. A., Siti Azizah. M.N., Yusri. Y., Norainy. M.H., Mohd Asnizam. A., Ali. A.B. (2002); year: 2017, 2018, 2019

##### Native status

Native species.

##### Conservation status

LC

#### Osteochilus scapularis

Fowler, 1939

7B1102DB-459F-5A52-84BE-8604EB6973B9

##### Materials

- **Type status:** Other material. **Occurrence:** catalogNumber: UMTZC8697; **Location:** locality: Saok River, Ikan River, Siput River; **Event:** samplingProtocol: Electrofishing; year: 2017, 2018, 2019

##### Native status

Native species.

##### Conservation status

LC

##### Notes

**Figure 10.**
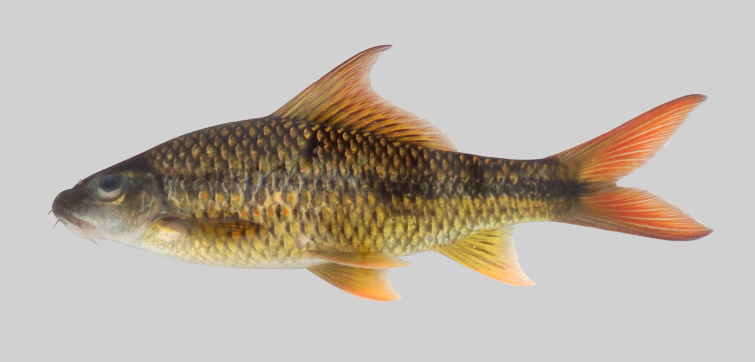
*Osteochilusscapularis*.

New record to Kenyir Reservoir (Fig. 10).

#### Osteochilus vittatus

(Valenciennes, 1842)

A287E595-9860-5331-AA9B-2C77694CCBFC

##### Materials

- **Type status:** Other material. **Occurrence:** catalogNumber: UMTZC7650; **Location:** locality: Cacing River, Lepar River, Lawit River, Siput River, Kiang River, Saok River, Cicir River, Perepek River, Mandak River; **Event:** samplingProtocol: Electrofishing, Literature; Yusoff. F. M., Zaidi. M. Z., Ambak. M. A. (1995); year: 2017, 2018, 2019, 2020

##### Native status

Native species.

##### Conservation status

LC

#### Osteochilus waandersii

(Bleeker, 1853)

298E7438-9DF7-5BA7-8BEE-C8B7E60788FD

##### Materials

- **Type status:** Other material. **Occurrence:** catalogNumber: UMTZC7663; **Location:** locality: Lepar River, Ikan River, Siput River, Saok River, Cacing River, Lawit River, Cicir River, Perepek River, Kiang River; **Event:** samplingProtocol: Electrofishing, Literature; Department of Fisheries (1994), Amirrudin. A., Siti Azizah. M.N., Yusri. Y., Norainy. M.H., Mohd Asnizam. A., Ali. A.B. (2002); year: 2017, 2018, 2019, 2020

##### Native status

Native species.

##### Conservation status

LC

#### Poropuntius normani

Smith, 1931

7497C799-8542-569A-9D33-5B5540ECE154

##### Materials

- **Type status:** Other material. **Occurrence:** catalogNumber: UMTZC7607; **Location:** locality: Lepar River, Kiang River, Ikan River, Saok River, Siput River, Pengait River, Cenana River; **Event:** samplingProtocol: Electrofishing, Literature; Zakaria. M.Z., Yaacob. K.K.K., Noor. J.M. (1997); year: 2017, 2018, 2019, 2020

##### Native status

Native species.

##### Conservation status

LC

#### Probarbus jullieni

Sauvage, 1880

9108CBE5-5EEB-533E-B71D-894191C2E87F

##### Materials

- **Type status:** Other material. **Event:** samplingProtocol: Literature; Kamaruddin. M.K.A., Mustafa-Kamal, A.S., Christianus. A., Daud. A., Abit. L.Y. (2011); Personal communication; Syed Muhammad Fuaad (2019)

##### Native status

Introduced species. The species was introduced into Kenyir Reservoir by the Department of Fisheries to increase the fisheries resources.

##### Conservation status

CR

#### Puntioplites bulu

(Bleeker, 1851)

32A2DDEE-3057-55F1-BE8B-4EBBAE4C248F

##### Materials

- **Type status:** Other material. **Occurrence:** catalogNumber: UMTZC8682; recordedBy: Aqmal-Naser. M., Ahmad. A.B. (unpublished); **Location:** locality: Mandak River; **Event:** samplingProtocol: Gill net; year: 2020

##### Native status

Native species.

##### Conservation status

LC

##### Notes

**Figure 11.**
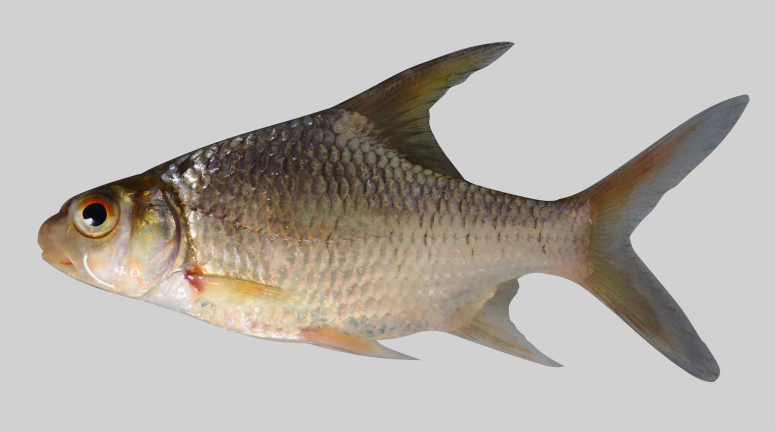
*Puntioplitesbulu*.

New record to Kenyir Reservoir (Fig. 11).

#### Tor tambra

(Valenciennes, 1842)

CDE5A29A-494E-5576-9CEB-45087AAD882A

##### Materials

- **Type status:** Other material. **Occurrence:** recordedBy: Ambak. M.A., Jalal. K.C.A.; **Location:** locality: Kiang River, Cenana River; **Event:** samplingProtocol: Electrofishing, Literature; Cramphorn. J. (1983); Department of Fisheries (1995); year: 2019

##### Native status

Native species.

##### Conservation status

DD

#### Esomus metallicus

Ahl, 1924

02203139-59DB-5F8E-821E-B0742E8404DA

##### Materials

- **Type status:** Other material. **Occurrence:** catalogNumber: UMTZC7131; **Location:** locality: Kiang River; **Event:** samplingProtocol: Electrofishing, Scoop net; year: 2017, 2019, 2020

##### Native status

Introduced species.

##### Notes

**Figure 12.**
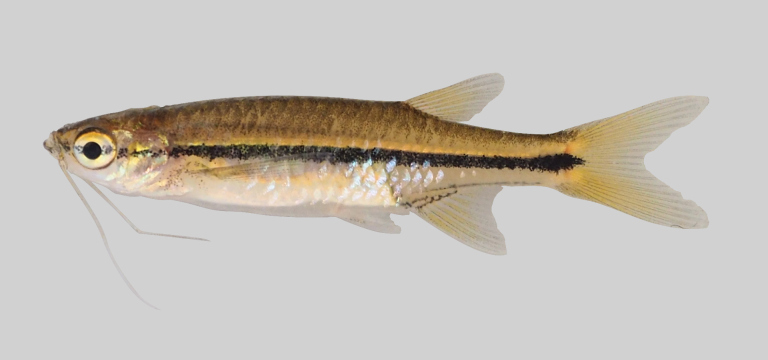
*Esomusmetallicus*.

New record to Kenyir Reservoir (Fig. 12). A common fish that thrives well in flood-plain or man-made habitats (Aqmal-Naser and Ahmad 2018a, Aqmal-Naser and Ahmad 2018b). Previously, this species is known as a native species in Peninsular Malaysia (Mohsin and Ambak 1983). However, it has been treated as an introduced species by Khan et al. (1996) and all recent studies without any justification. The identity of this species will soon be determined and the article on its status is being prepared. Hence, at the moment, we regarded this species as introduced species.

#### Raiamas guttatus

(Day, 1870)

BF37A350-265B-5D2D-BB54-87892DE60E5C

##### Materials

- **Type status:** Other material. **Event:** samplingProtocol: Literature; Ambak. M.A., Jalal. K.C.A (1998)

##### Native status

Native species.

##### Conservation status

LC

#### Rasbora myersi

Brittan, 1954

30147789-543B-5F5A-8700-1502E893A444

##### Materials

- **Type status:** Other material. **Occurrence:** catalogNumber: UMTZC7635; **Location:** locality: Lepar River, Saok River, Kiang River, Lawit River, Cicir River, Perepek River, Mandak River, Siput River; **Event:** samplingProtocol: Electrofishing, Literature; Yusoff. F.M., Zaidi. M.Z., Ambak. M.A. (1995); year: 2017, 2019, 2020

##### Native status

Native species

##### Conservation status

LC

#### Rasbora notura

Kottelat, 2005

5390FCAE-7BC5-5F44-A6D8-A94A5ED7DE30

##### Materials

- **Type status:** Other material. **Occurrence:** catalogNumber: UMTZC7675; **Location:** locality: Ikan River; **Event:** samplingProtocol: Electrofishing, Literature; Amirrudin. A., Siti Azizah. M.N., Yusr.i Y., Norainy. M.H., Mohd Asnizam. A., Ali. A.B. (2002); year: 2017, 2018, 2019, 2020

##### Native status

Native species.

##### Conservation status

LC

#### Rasbora sp. 1

FD867C63-B1E5-5333-8959-428DB248D994

##### Materials

- **Type status:** Other material. **Occurrence:** catalogNumber: UMTZC7675; **Location:** locality: Ikan River, Kiang River; **Event:** samplingProtocol: Electrofishing; year: 2019, 2019

##### Native status

Native species.

##### Conservation status

NE

##### Notes

**Figure 13.**
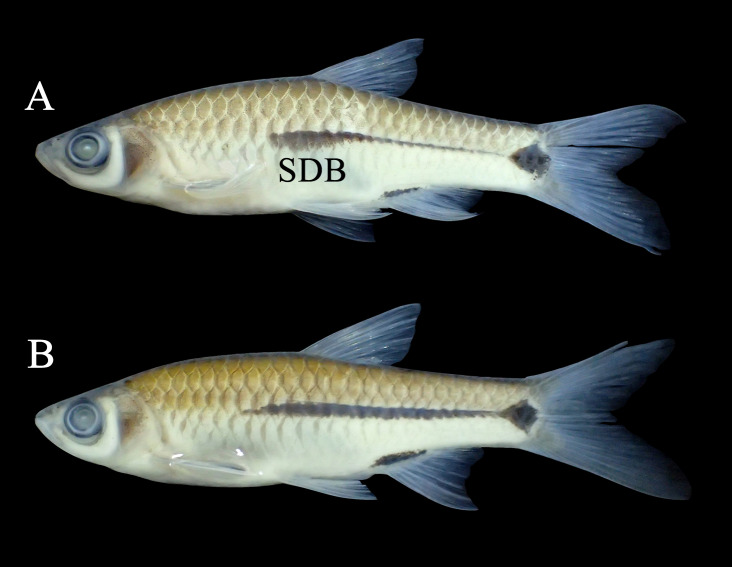
*Rasboranotura* (A) and *Rasbora* sp. 1 (B). SDB: Subdorsal blotch.

New record to Kenyir Reservoir. The *Rasbora* sp. 1 in this study has a thicker and anteriorly tapered mid-lateral stripe and the subdorsal blotch is absent, which did not fit into the description of *Rasboranotura* by Kottelat (2005) (Fig. 13).

#### Rasbora paucisqualis

Ahl, 1935

92B94632-EF07-5120-BB23-C3773FC78E9F

##### Materials

- **Type status:** Other material. **Occurrence:** catalogNumber: UMTZC8683; **Location:** locality: Papan River; **Event:** samplingProtocol: Electrofishing; year: 2020

##### Native status

Native species.

##### Conservation status

LC

##### Notes

**Figure 14.**
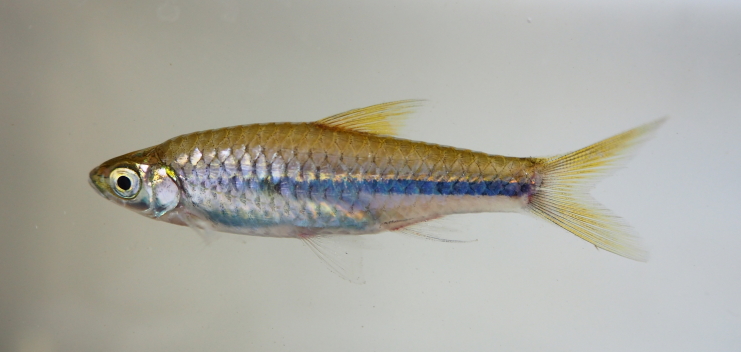
*Rasborapaucisqualis*.

New record to Kenyir Reservoir (Fig. 14).

#### Rasbora paviana

Tirant, 1885

76EFFCDF-EE19-5B1E-A796-E67D449E46F5

##### Materials

- **Type status:** Other material. **Occurrence:** catalogNumber: UMTZC7647; **Location:** locality: Ikan River, Saok River, Kiang River, Lawit River, Siput River; **Event:** samplingProtocol: Electrofishing, Literature; Department of Fisheries (1994); year: 2017, 2018, 2019, 2020

##### Native status

Native species.

##### Conservation status

LC

#### Rasbora sp. 2

BFCC5A95-ABFD-5725-86D3-50884FD7035D

##### Materials

- **Type status:** Other material. **Occurrence:** catalogNumber: UMTZC7648; **Location:** locality: Ikan River, Papan River; **Event:** samplingProtocol: Electrofishing; year: 2018, 2019, 2020

##### Native status

Native species.

##### Conservation status

NE

##### Notes

**Figure 15.**
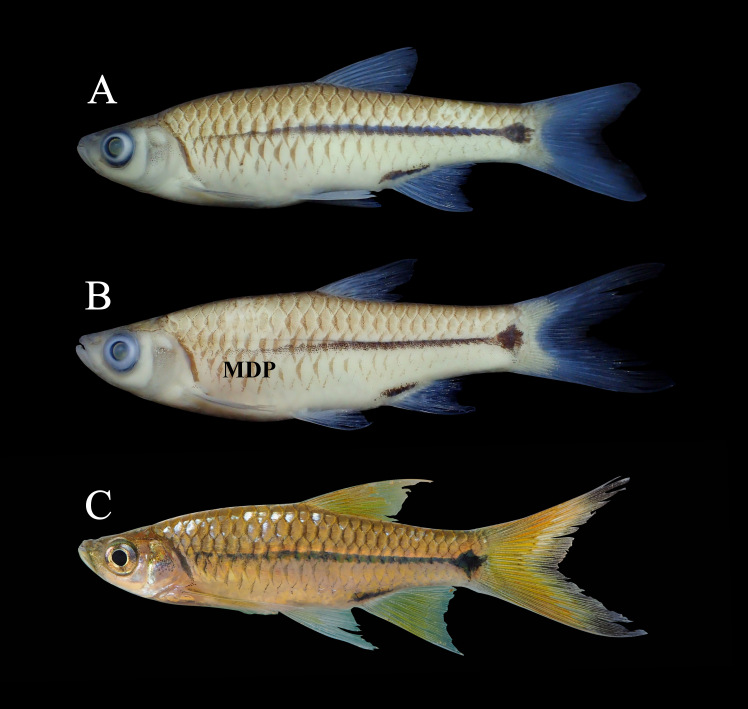
*Rasborapaviana* (A) and *Rasbora* sp. 2 (B & C). Mid-humeral diffuse patch (MDP).

New record to Kenyir Reservoir. There were two forms of *Rasbora* sp. collected in this study. A more common form has an incomplete mid-lateral stripe (not reaching gill opening) which is different from the real *Rasborapaviana* (Fig. 15A). It has the mid-lateral line that begins to diffuse at the mid-section of the body, known as a mid-humeral diffuse patch (MDP) (Lumbantobing 2014) (Fig. 15B). One specimen displayed a unique set of characteristics: high and pointed dorsal fin (vs. rounded), long and, when adpressed, reaching beyond the base of the anal fin (vs. only half); pectoral fin long and overlapping pelvic fin (vs. not reaching the pelvic fin); pelvic fin elongated and reaching half of the dorsal fin base (vs. not overlapping) and possessing diamond-shape caudle peduncle blotch (vs. longitudinally elongated diamond) (Fig. 15C). Due to a lack of specimens, a detailed comparison was not made at this moment.

#### Leptobarbus rubripinna

(Fowler, 1937)

6D3B0DE5-7CFA-5056-8A46-C40C31D6E7E5

##### Materials

- **Type status:** Other material. **Location:** locality: Lawit River; **Event:** samplingProtocol: Gill net, Literature; Yusoff. F.M., Zaidi. M.Z., Ambak. M.A. (1995); year: 2019

##### Native status

Introduced species. The species was introduced into Kenyir Reservoir through the restocking programme in 1988-1990 by the Department of Fisheries.

#### Hypophthalmichthys nobilis

(Richardson, 1845)

5D784CA2-F4A1-59AF-B979-1B7A56F60273

##### Materials

- **Type status:** Other material. **Event:** samplingProtocol: Literature; Yusoff. F.M., Zaidi. M.Z., Ambak. M.A. (1995)

##### Native status

Introduced species. The species was introduced into Kenyir Reservoir through the restocking programme in 1988-1990 by the Department of Fisheries.

#### Oxygaster anomalura

van Hasselt, 1823

C92FA3BD-060F-5C2E-9E02-C13B70A75155

##### Materials

- **Type status:** Other material. **Occurrence:** catalogNumber: UMTZC7690; **Location:** locality: Cicir River; **Event:** samplingProtocol: Electrofishing, Literature; Yusoff. F.M., Zaidi. M.Z., Ambak. M.A. (1995); year: 2019

##### Native status

Native species.

##### Conservation status

LC

#### Piaractus brachypomus

(Cuvier, 1818)

C6E8E3F9-1439-557E-8CAC-CEBFB70496F4

##### Materials

- **Type status:** Other material. **Occurrence:** recordedBy: Yusoff. F.M., Zaidi. M.Z., Ambak. M.A. (1995); **Event:** samplingProtocol: Literature

##### Native status

Introduced species used for recreational cage culture in Kenyir Reservoir.

#### Hemibagrus capitulum

(Popta, 1906)

FC849774-7835-5EB6-8703-1A5C82EE3796

##### Materials

- **Type status:** Other material. **Occurrence:** catalogNumber: UMTZC7637; **Location:** locality: Lepar River, Ikan River, Siput River, Saok River, Cicir River, Cenana River, Pengait River; **Event:** samplingProtocol: Electrofishing, Literature; Ambak. M.A., Jalal. K.C.A (1998); year: 2017, 2019, 2020

##### Native status

Native species.

##### Conservation status

LC

#### Hemibagrus gracilis

Ng and Ng, 1995

1BD39B8E-625B-558A-AC6A-C405D3864EC0

##### Materials

- **Type status:** Other material. **Occurrence:** catalogNumber: UMTZC7631; **Location:** locality: Siput River, Lepar River; **Event:** samplingProtocol: Electrofishing, Literature; Cramphorn. J. (1983); year: 2017, 2019

##### Native status

Native species.

##### Conservation status

LC

#### Hemibagrus wyckii

(Bleeker, 1858)

13E8246C-E616-5A9B-AD78-BB5CBCC98748

##### Materials

- **Type status:** Other material. **Event:** samplingProtocol: Literature; Cramphorn. J. (1983); Department of Fisheries (1994)

##### Native status

Native species.

##### Conservation status

LC

#### Mystus castaneus

Ng, 2002

E88D9CEB-30F1-57C0-825E-FA7BAA430AEB

##### Materials

- **Type status:** Other material. **Occurrence:** catalogNumber: UMTZC8684; **Location:** locality: Budu River; **Event:** samplingProtocol: Electrofishing; year: 2020

##### Native status

Native species.

##### Conservation status

LC

#### Mystus singaringan

(Bleeker, 1846)

66003E76-6DAA-5564-912D-8E8FDDDFBBFF

##### Materials

- **Type status:** Other material. **Occurrence:** catalogNumber: UMTZC8685; **Location:** locality: Ikan River,; **Event:** samplingProtocol: Electrofishing; year: 2020

##### Native status

Native species.

##### Conservation status

LC

##### Notes

**Figure 16.**
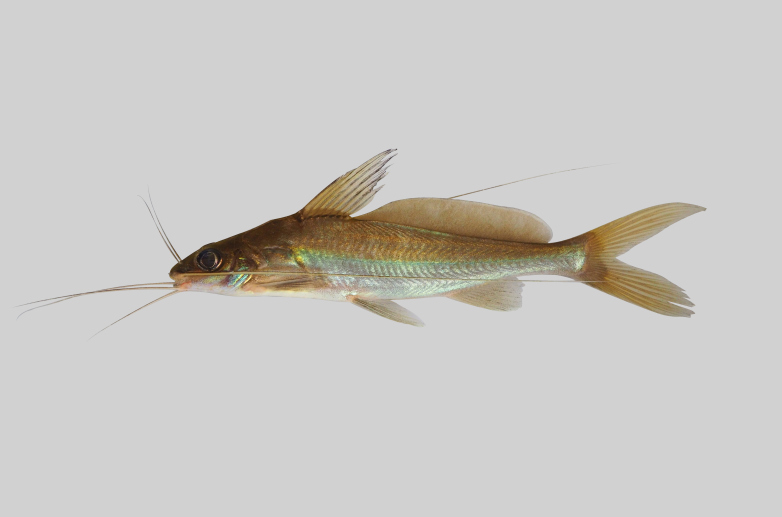
*Mystussingaringan*.

New record to Kenyir Reservoir (Fig. 16).

#### Amblyceps foratum

Ng and Kottelat, 2000

C605DB6A-7E2F-5BCF-8BA1-0B34953922FF

##### Materials

- **Type status:** Other material. **Occurrence:** catalogNumber: UMTZC7711; **Location:** locality: Kiang River, Siput River; **Event:** samplingProtocol: Electrofishing; year: 2017, 2019

##### Native status

Native species.

##### Conservation status

LC

##### Notes

**Figure 17.**
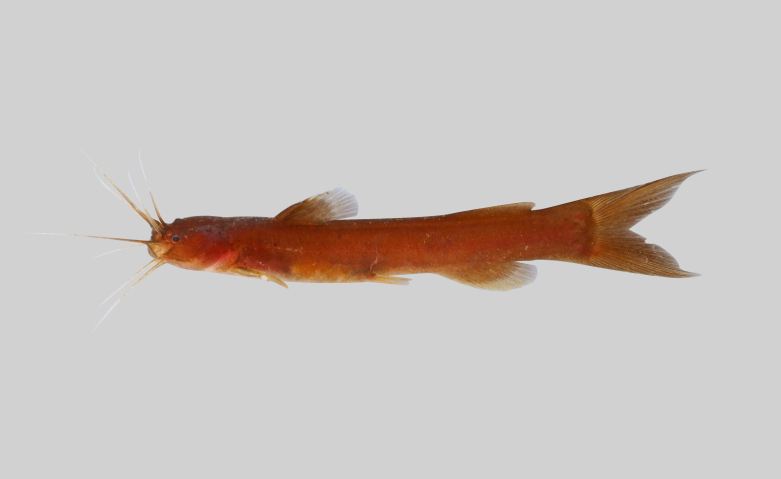
*Amblycepsforatum*.

New record to Kenyir Reservoir (Fig. 17).

#### Glyptothorax fuscus

Fowler, 1934

5A18A064-F7C6-53BE-91E1-97E1CB5610F6

##### Materials

- **Type status:** Other material. **Occurrence:** catalogNumber: UMTZC8687; **Location:** locality: Perepek River, Kiang River, Siput River; **Event:** samplingProtocol: Electrofishing, Literature; Cramphorn. J. (1983); year: 2017

##### Native status

Native species.

##### Conservation status

LC

#### Glyptothorax schmidti

(Volz, 1904)

25066C16-0B2C-566A-9383-01C917FAFB59

##### Materials

- **Type status:** Other material. **Occurrence:** catalogNumber: UMTZC7705; **Location:** locality: Siput River; **Event:** samplingProtocol: Electrofishing; year: 2017, 2018, 2019

##### Native status

Native species.

##### Conservation status

LC

##### Notes

**Figure 18.**
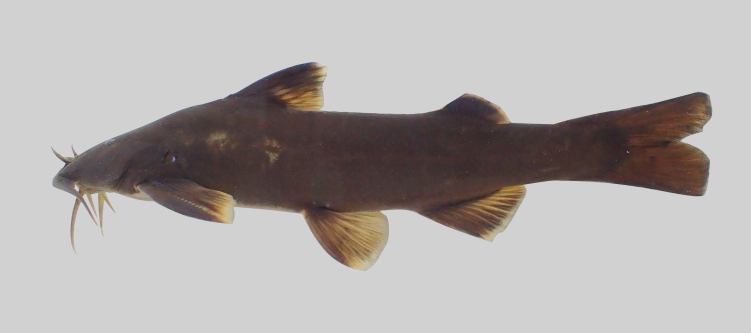
*Glyptothoraxschmidti*.

New record to Kenyir Reservoir (Fig. 18).

#### Pangasianodon hypophthalmus

(Sauvage, 1878)

63A9C1B1-5249-58D2-8E0F-EB09DAA778BA

##### Materials

- **Type status:** Other material. **Location:** locality: Lawit River; **Event:** samplingProtocol: Literature; Shahid. S.M., Fairuz. M.S.M., Jamil. Z.A., Radzali. M.M., Hariz. A.R.M., Fahimee. J.M., Wira. A.B., Zafrul. A.R.M., Hasliana. K. (2010) , Personal communication; Syed Muhammad Fuaad; year: 2020

##### Native status

Introduced species. The species was introduced through the restocking programme to increase the fisheries resources.

#### Pangasius nasutus

(Bleeker, 1863)

CF6B1274-F00C-583B-AC88-B55427425D98

##### Materials

- **Type status:** Other material. **Event:** samplingProtocol: Literature; Yusoff. F.M., Zaidi. M.Z., Ambak. M.A. (1995)

##### Native status

Introduced species used for recreational cage culture in Kenyir Reservoir.

##### Conservation status

LC

#### Ompok siluroides

Lacepède, 1803

1ABD42A1-EFF3-5DA2-AA25-F229DA7370CE

##### Materials

- **Type status:** Other material. **Occurrence:** catalogNumber: UMTZC7782; **Location:** locality: Lawit River, Kiang River; **Event:** samplingProtocol: Electrofishing; year: 2017, 2019

##### Native status

Native species

##### Conservation status

LC

#### Wallagonia leerii

(Bleeker, 1851)

EA0E8D97-F842-5043-BDC4-2452F3864EE0

##### Materials

- **Type status:** Other material. **Event:** samplingProtocol: Literature; Ambak. M.A., Jalal. K.C.A. (1998)

##### Native status

Native species.

##### Conservation status

LC

#### Clarias aff. batrachus

(Linnaeus, 1758)

476DCB97-E616-5E18-AD0B-BAF3AABC2310

##### Materials

- **Type status:** Other material. **Occurrence:** catalogNumber: UMTZC7792; **Location:** locality: Mandak River; **Event:** samplingProtocol: Electrofishing, Literature; Department of Fisheries (1995); year: 2019

##### Native status

Native species.

##### Conservation status

NE

##### Notes

**Figure 19.**
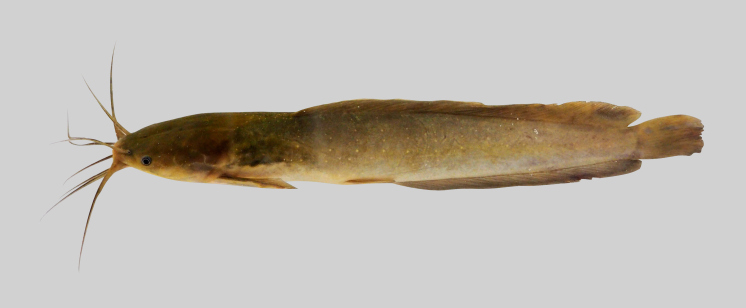
Clariasaff.batrachus from Kenyir Reservoir.

The distribution of *Clariasbatrachus* is restricted to Java, Indonesia (Ng and Kottelat 2008). The species is widely introduced for cultivation, but originated from an Indochinese subpopulation that may represent undescribed species (Ng and Low 2019). The previously-identified population of *C.batrachus* in north-eastern India is presently known as *Clariasmagur*. Other populations were known as Clariasaff.batrachus ‘Indochina’ (from Mekong River drainage) and Clariasaff.batrachus ‘Sundaland’ (from the Malay Peninsula and Borneo) (Fig. 19).

#### Clarias leiacanthus

Bleeker, 1851

C6BD16B8-90F6-5FD5-9FF6-6354C0E777F1

##### Materials

- **Type status:** Other material. **Occurrence:** catalogNumber: UMTZC7750; **Location:** locality: Ikan River, Kiang River, Lawit River; **Event:** samplingProtocol: Electrofishing; year: 2017, 2018, 2019

##### Native status

Native species.

##### Conservation status

LC

##### Notes

**Figure 20.**
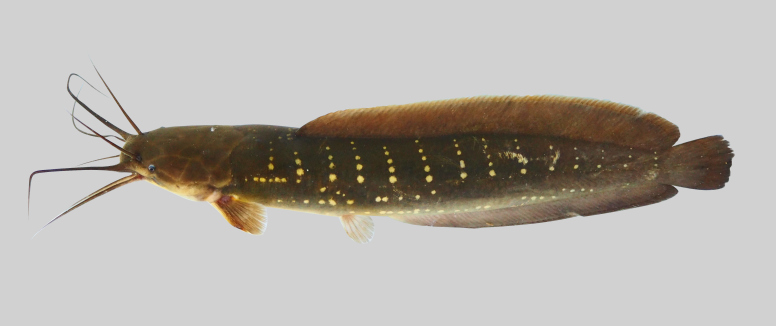
*Clariasleiacanthus*.

New record to Kenyir Reservoir (Fig. 20).

#### Oxyeleotris marmorata

(Bleeker, 1852)

3346A8FE-0FCD-53A4-A9AA-4068DE4E2665

##### Materials

- **Type status:** Other material. **Occurrence:** catalogNumber: UMTZC7760; **Location:** locality: Kiang River, Siput River, Lepar River, Cicir River, Perepek River, Mandak River; **Event:** samplingProtocol: Electrofishing, Literature; Ambak. M.A., Jalal. K.C.A. (1998); year: 2017, 2018, 2019, 2020

##### Native status

Native species.

##### Conservation status

LC

#### Glossogobius giuris

(Hamilton, 1822)

9FD94827-AD8F-56DE-BD4F-8D0779274F55

##### Materials

- **Type status:** Other material. **Occurrence:** catalogNumber: UMTZC7386; **Location:** locality: Siput River; **Event:** samplingProtocol: Electrofishing; year: 2019

##### Native status

Native species.

##### Conservation status

LC

##### Notes

**Figure 21.**
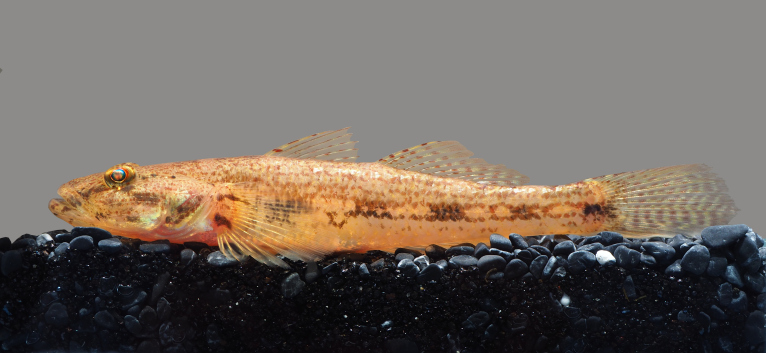
*Glossogobiusgiuris*.

New record to Kenyir Reservoir (Fig. 21).

#### Pseudogobiopsis oligactis

(Bleeker, 1875)

F17FF41A-9DC7-57C5-8873-7E54B1970426

##### Materials

- **Type status:** Other material. **Occurrence:** catalogNumber: UMTZC7696; **Location:** locality: Ikan River, Kiang River, Siput River, Lepar River, Cicir River, Perepek River, Mandak River, Cenana River; **Event:** samplingProtocol: Electrofishing, Literature; Ambak. M.A., Jalal. K.C.A. (1998); year: 2017, 2018, 2019, 2020

##### Native status

Native species.

##### Conservation status

LC

#### Macrognathus circumcinctus

(Hora, 1924)

DC6C9C05-5BD0-5002-AACB-21453015FBAA

##### Materials

- **Type status:** Other material. **Occurrence:** catalogNumber: UMTZC8688; **Location:** locality: Kiang River; **Event:** samplingProtocol: Electrofishing; year: 2017, 2019

##### Native status

Native species.

##### Conservation status

LC

##### Notes

**Figure 22.**
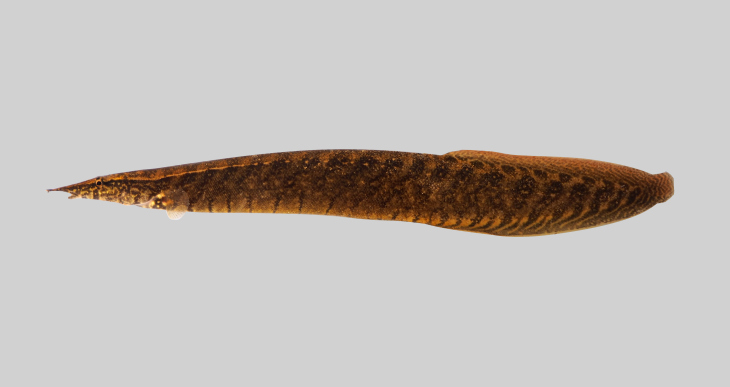
*Macrognathuscircumcinctus*.

New record to Kenyir Reservoir (Fig. 22).

#### Mastacembelus favus

Hora, 1923

9910B837-D7E5-5CC7-9EBC-3E0FE433F371

##### Materials

- **Type status:** Other material. **Occurrence:** catalogNumber: UMTZC7694; **Location:** locality: Siput River, Kiang River, Mandak River; **Event:** samplingProtocol: Electrofishing, Literature; Ambak. M.A., Jalal. K.C.A. (1998); year: 2017, 2019

##### Native status

Native species.

##### Conservation status

LC

#### Mastacembelus unicolor

Cuvier, 1832

3D4867B3-D8C1-5086-A79F-0D671301E21C

##### Materials

- **Type status:** Other material. **Occurrence:** catalogNumber: UMTZC8689; **Location:** locality: Kiang River; **Event:** samplingProtocol: Electrofishing; year: 2017

##### Native status

Native species.

##### Conservation status

LC

##### Notes

**Figure 23.**
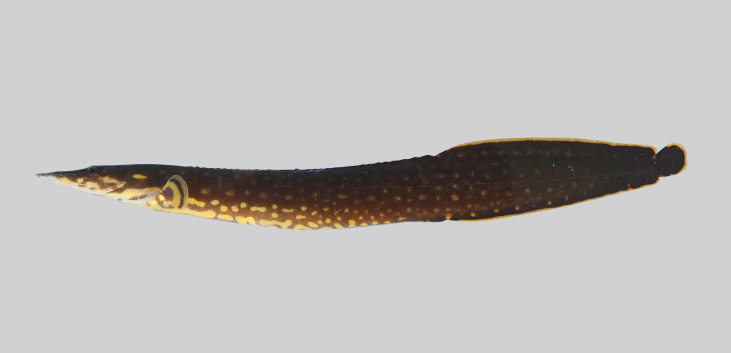
*Mastacembelusunicolor*.

New record to Kenyir Reservoir (Fig. 23).

#### Monopterus javanensis

Lacepède, 1800

DF53E77F-90F2-5DE5-9DFA-55C2B169205E

##### Materials

- **Type status:** Other material. **Occurrence:** catalogNumber: UMTZC8690; **Location:** locality: Mandak River, Kiang River; **Event:** samplingProtocol: Electrofishing; year: 2018, 2019

##### Native status

Native species.

##### Conservation status

LC

##### Notes

**Figure 24.**
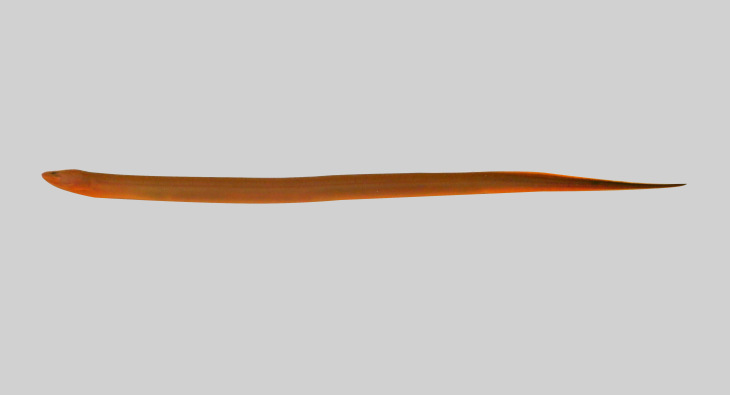
*Monopterusjavanensis*.

New record to Kenyir Reservoir (Fig. 24).

#### Betta stigmosa

Tan and Ng, 2005

A658E3D6-3F14-5465-A1C1-BE1D08A8E45C

##### Materials

- **Type status:** Other material. **Occurrence:** catalogNumber: UMTZC8691; **Location:** locality: Ikan River; **Event:** samplingProtocol: Electrofishing, Scoop net; year: 2017, 2018, 2019

##### Native status

Native species.

##### Conservation status

DD

##### Notes

**Figure 25.**
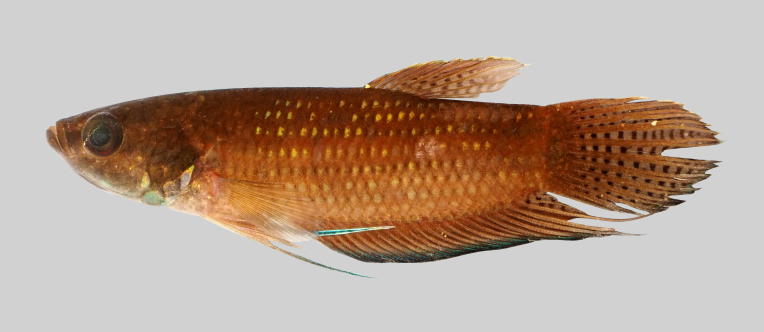
*Bettastigmosa*.

New record to Kenyir Reservoir (Fig. 25).

#### Osphronemus goramy

Lacepède, 1801

D993FFAA-56E2-5F41-90EC-3A0CB12EECD3

##### Materials

- **Type status:** Other material. **Occurrence:** catalogNumber: UMTZC8692; **Location:** locality: Saok River; **Event:** samplingProtocol: Electrofishing, Literature; Department of Fisheries (1994); Ambak. M.A., Jalal. K.C.A. (1998); year: 2018, 2019

##### Native status

Native species.

##### Conservation status

LC

#### Trichopodus trichopterus

(Pallas, 1770)

A6316F6B-C380-5593-A167-006C0C1AEE34

##### Materials

- **Type status:** Other material. **Occurrence:** catalogNumber: UMTZC7636; **Location:** locality: Siput River, Cicir River; **Event:** samplingProtocol: Electrofishing; year: 2019, 2020

##### Native status

Native species.

##### Conservation status

LC

##### Notes

**Figure 26.**
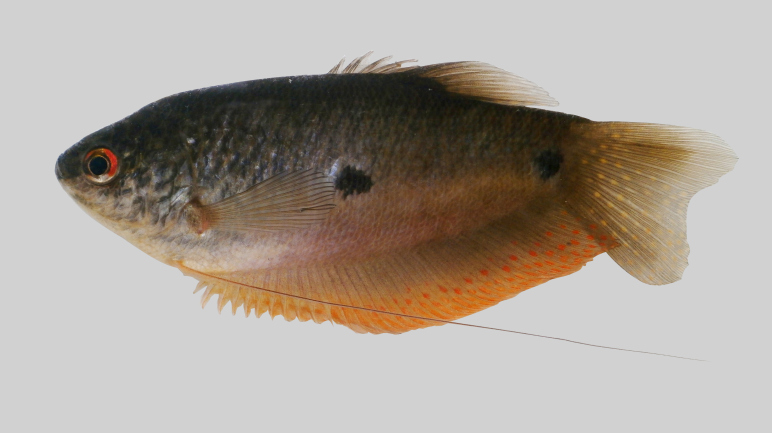
*Trichopodustrichopterus*.

New record to Kenyir Reservoir (Fig. 26).

#### Trichopsis vittata

(Cuvier, 1831)

C56192A8-9A46-52BF-95A1-8BCEACA87752

##### Materials

- **Type status:** Other material. **Occurrence:** catalogNumber: UMTZC8693; **Location:** locality: Kiang River; **Event:** samplingProtocol: Electrofishing, Scoop net; year: 2019, 2020

##### Native status

Native species.

##### Conservation status

LC

##### Notes

**Figure 27.**
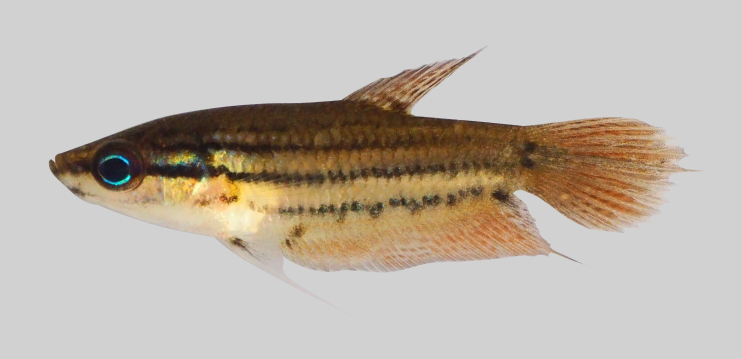
*Trichopsisvittata*.

New record to Kenyir Reservoir (Fig. 27).

#### Channa limbata

(Cuvier, 1831)

43E1DCBE-E093-51BB-9E7E-F4C59F90A921

##### Materials

- **Type status:** Other material. **Occurrence:** catalogNumber: UMTZC8694; **Location:** locality: Saok River; **Event:** samplingProtocol: Electrofishing, Scoop net; year: 2020

##### Native status

Native species.

##### Conservation status

LC

##### Notes

**Figure 28.**
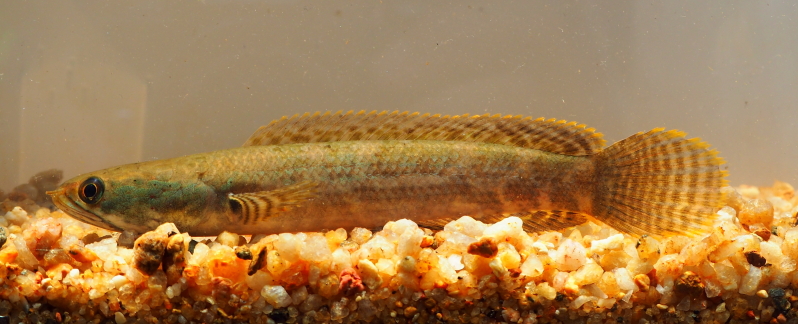
*Channalimbata*.

New record to Kenyir Reservoir (Fig. 28).

#### Channa lucius

(Cuvier, 1831)

427C8464-A3D7-52AD-A27C-C8CBC25439FC

##### Materials

- **Type status:** Other material. **Occurrence:** recordedBy: Fahmi-Ahmad. M., Walton. S. (unpublished); **Location:** locality: Cacing River; **Event:** samplingProtocol: Gill net,; year: 2014

##### Native status

Native species.

##### Conservation status

LC

#### Channa marulioides

(Bleeker, 1851)

51E48ED8-C60F-59CD-8E63-388FA5485F65

##### Materials

- **Type status:** Other material. **Event:** samplingProtocol: Literature; Department of Fisheries (1995)

##### Native status

Native species.

##### Conservation status

LC

#### Channa micropeltes

(Cuvier, 1831)

C110B6D3-B3B6-5C9E-B835-7DCD58DD9EE0

##### Materials

- **Type status:** Other material. **Location:** locality: Cacing River; **Event:** samplingProtocol: Electrofishing, Literature; Ambak. M.A., Jalal. K.C.A (1998); year: 2019, 2020

##### Native status

Native species.

##### Conservation status

LC

#### Channa striata

(Bloch, 1793)

E467176C-3CD3-502B-A81E-230AE0C4138C

##### Materials

- **Type status:** Other material. **Occurrence:** catalogNumber: UMTZC7654; **Location:** locality: Siput River, Mandak River; **Event:** samplingProtocol: Electrofishing, Literature; Ambak. M.A., Jalal. K.C.A (1998); year: 2017, 2018, 2019

##### Native status

Native species.

##### Conservation status

LC

#### Pristolepis grootii

(Bleeker, 1852)

6346AD02-AF6B-54DE-9FAC-FCBB874FDA2C

##### Materials

- **Type status:** Other material. **Occurrence:** catalogNumber: UMTZC7695; **Location:** locality: Lepar River, Siput River, Kiang River, Mandak River; **Event:** samplingProtocol: Electrofishing, Literature; Department of Fisheries (1994); year: 2017, 2018, 2019, 2020

##### Native status

Native species.

##### Conservation status

LC

#### Lates calcarifer

(Bloch, 1790)

808D2DFD-F7D9-5209-8AB1-B37F5EEDC9C7

##### Materials

- **Type status:** Other material. **Event:** samplingProtocol: Literature; Yusoff. F.M., Zaidi. M.Z., Ambak. M. (1995)

##### Native status

Introduced species for sports fishing in the Kenyir Reservoir.

#### Oreochromis niloticus

(Linnaeus, 1758)

63A2A962-77F8-53FE-9687-AF5F0919D45A

##### Materials

- **Type status:** Other material. **Event:** samplingProtocol: Literature; Department of Fisheries (1995)

##### Native status

Introduced species.

#### Gambusia affinis

(Baird and Girard, 1853)

C0A7D226-3919-53AE-B882-C3466190AD1B

##### Materials

- **Type status:** Other material. **Occurrence:** catalogNumber: UMTZC8696; **Location:** locality: Saok River; **Event:** samplingProtocol: Electrofishing,; year: 2017, 2018, 2019

##### Native status

Introduced species. Probably introduced via human-mediated translocation.

#### Xenentodon canciloides

(Bleeker, 1854)

A03CACEE-A5FB-5596-AED5-766B4599FFCF

##### Materials

- **Type status:** Other material. **Occurrence:** catalogNumber: UMTZC7741; **Location:** locality: Cacing River; **Event:** samplingProtocol: Electrofishing, Gills net; year: 2014

##### Native status

Native species.

##### Conservation status

LC

##### Notes

**Figure 29.**
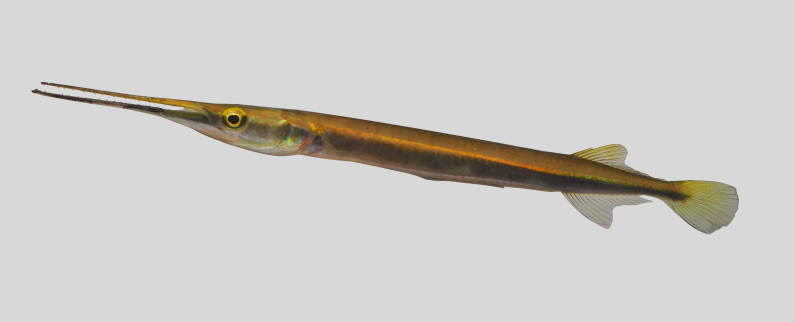
*Xenentodoncanciloides*.

New record to Kenyir Reservoir (Fig. 29).

#### Pao leiurus

(Bleeker, 1850)

73E77CA0-D115-5417-8DEF-C8B7AD2FCE9C

##### Materials

- **Type status:** Other material. **Occurrence:** catalogNumber: UMTZC7716; **Location:** locality: Siput River; **Event:** samplingProtocol: Electrofishing, Literature; Ambak. M.A., Jalal. K.C.A (1998); year: 2017

##### Native status

Native species.

##### Conservation status

LC

#### Parambassis siamensis

(Fowler, 1937)

93FE7EA0-DD72-59E6-835D-696E25FC4F7B

##### Materials

- **Type status:** Other material. **Occurrence:** catalogNumber: UMTZC7785; **Location:** locality: Cicir River, Perepek River, Kiang River, Mandak River; **Event:** samplingProtocol: Electrofishing; year: 2017, 2018, 2019, 2020

##### Native status

Native species.

##### Conservation status

LC

## Discussion

The species recorded contributed 25.95% of the total 289 freshwater fishes of Peninsular Malaysia (Zakaria-Ismail et al. 2019). We expect more fish species can be recorded by increasing the sampling efforts, since several families and species were already reported within the Terengganu River Basins, such as Balitoridae (*Homalopteraparclitella*), Nemacheilidae (*Nemacheilusselangoricus*), Xenocyprididae (*Parachela* spp.), Bagridae (*Leiocassispoeciloptera* and *Pseudomystus* spp.), Syngnathidae (*Doryichthys* spp.) and Akysidae (*Akysis* spp. & *Acrochordonichthys* spp.) (Aqmal-Naser et al. in press), but currently were not recorded in Kenyir Reservoir.

In his study, Cramphorn (1983) recorded the presence of *Mystacoleucuschilopterus*, but the post-inundation surveys failed to record this species. This species could have been present before, but was diminished or reduced as a result of impoundment or we did not hit the right spot to collect this species. This species is a common species found in the larger rivers, especially in the eastern part of Peninsular Malaysia (Zakaria-Ismail et al. 2019). Other species, such as *Barbichtyhslaevis* and *Wallagonialeerii*, were only recorded during the early post-inundation (Yusoff et al. 1995, Ambak and Jalal 1998). It was known that *Barbichthyslaevis* cannot cope with the impoundment (Rainboth 1996), while *Wallagonialeerii* is a highly migratory species Riede (2004) where the impoundment could have impacted the migratory pathway for these species. Further assessments are needed to confirm the presence of these species through comprehensive field sampling. One species, *Scleropagesformosus*, is now facing the threat for its highly ornamental values.

More studies also are encouraged to be done on other species especially in the Data Deficient category especially *Tortambra* (Walton et al. 2016) and *Bettastigmosa* (Low 2019) for the updated status on their distribution and populations. We also did find any species in the ghost nets in the streams that are vulnerable to migratory fish species especially, cyprinids fish, concurrent with the finding of Aqmal-Naser and Ahmad (2020), which found dead *Tortambra* entangled in the ghost net. Nevertheless, it is very difficult to monitor or control the fishing activities in Kenyir Reservoir due to its massive waterbody and limited enforcement.

There were three native species which were introduced for fisheries enrichment (i.e. *Pangasiusnasutus* and *Probarbusjullieni*) and sports fishing (i.e. *Latescalcarifer*). Both *P.jullieni* and *Latescalcarifer* are thriving well in the lentic ecosystem of Kenyir Reservoir and were collected by the local people for artisanal fisheries but no information regarding *Pangasiusnasutus* till now. The mosquito-fish, *Gambusiaaffinis* is the most widespread especially in the human-associated area. It is unknown how this species ended up in Kenyir Reservoir, but probably a result of aquarium dumping, one of the major practices which lead to the alien fish species introduction (Aqmal-Naser and Ahmad 2020). However, other introduced species are rarely seen or collected, which sparks a debate on their ability to survive. For example, the juveniles of *Barbonymusgonionotus* and *Hypopthalmichthysnobilis* have been released by the Department of Fisheries (Yusoff et al. (1995), but they have never been reported or collected till the present day. The fish could be present and caught by the local fisherman, but there are no documentation and records.

All six fish species highlighted required taxonomic revision and molecular evidence to elucidate their true taxonomic lineage. The cryptic diversity amongst freshwater fishes, especially from the genus *Rasbora*, is one of the interesting subjects to begin with. We recommend more studies that integrate both morphology and the molecular aspect to resolve the taxonomic ambiguity of freshwater fishes, generally. The new fish records in Kenyir Reservoir revealed its importance as one of the conservation areas in Peninsular Malaysia. The presence of successful alien fish species like *Gambusiaaffinis* which can have negative impacts on the native aquatic fauna should be monitored.

## Supplementary Material

XML Treatment for Scleropages formosus

XML Treatment for Chitala cf. lopis

XML Treatment for Notopterus notopterus

XML Treatment for Clupeichthys sp.

XML Treatment for Acantopsis dialuzona

XML Treatment for Acanthopsoides molobrion

XML Treatment for Pangio doriae

XML Treatment for Pangio filinaris

XML Treatment for Pangio semicincta

XML Treatment for Balitoropsis zollingeri

XML Treatment for Homaloptera ogilviei

XML Treatment for Homalopteroides tweediei

XML Treatment for Pseudohomaloptera leonardi

XML Treatment for Nemacheilus masyae

XML Treatment for Barbichthys laevis

XML Treatment for Barbodes rhombeus

XML Treatment for Barbodes sellifer

XML Treatment for Barbodes lateristriga

XML Treatment for Barbonymus gonionotus

XML Treatment for Barbonymus schwanefeldii

XML Treatment for Crossocheilus oblongus

XML Treatment for Cyclocheilichthys apogon

XML Treatment for Cyclocheilichthys armatus

XML Treatment for Cyprinus carpio

XML Treatment for Ceratogarra cambodgiensis

XML Treatment for Hampala macrolepidota

XML Treatment for Labiobarbus leptocheilus

XML Treatment for Lobocheilos rhabdoura

XML Treatment for Mystacoleucus chilopterus

XML Treatment for Mystacoleucus obtusirostris

XML Treatment for Neolissochilus soroides

XML Treatment for Osteochilus scapularis

XML Treatment for Osteochilus vittatus

XML Treatment for Osteochilus waandersii

XML Treatment for Poropuntius normani

XML Treatment for Probarbus jullieni

XML Treatment for Puntioplites bulu

XML Treatment for Tor tambra

XML Treatment for Esomus metallicus

XML Treatment for Raiamas guttatus

XML Treatment for Rasbora myersi

XML Treatment for Rasbora notura

XML Treatment for Rasbora sp. 1

XML Treatment for Rasbora paucisqualis

XML Treatment for Rasbora paviana

XML Treatment for Rasbora sp. 2

XML Treatment for Leptobarbus rubripinna

XML Treatment for Hypophthalmichthys nobilis

XML Treatment for Oxygaster anomalura

XML Treatment for Piaractus brachypomus

XML Treatment for Hemibagrus capitulum

XML Treatment for Hemibagrus gracilis

XML Treatment for Hemibagrus wyckii

XML Treatment for Mystus castaneus

XML Treatment for Mystus singaringan

XML Treatment for Amblyceps foratum

XML Treatment for Glyptothorax fuscus

XML Treatment for Glyptothorax schmidti

XML Treatment for Pangasianodon hypophthalmus

XML Treatment for Pangasius nasutus

XML Treatment for Ompok siluroides

XML Treatment for Wallagonia leerii

XML Treatment for Clarias aff. batrachus

XML Treatment for Clarias leiacanthus

XML Treatment for Oxyeleotris marmorata

XML Treatment for Glossogobius giuris

XML Treatment for Pseudogobiopsis oligactis

XML Treatment for Macrognathus circumcinctus

XML Treatment for Mastacembelus favus

XML Treatment for Mastacembelus unicolor

XML Treatment for Monopterus javanensis

XML Treatment for Betta stigmosa

XML Treatment for Osphronemus goramy

XML Treatment for Trichopodus trichopterus

XML Treatment for Trichopsis vittata

XML Treatment for Channa limbata

XML Treatment for Channa lucius

XML Treatment for Channa marulioides

XML Treatment for Channa micropeltes

XML Treatment for Channa striata

XML Treatment for Pristolepis grootii

XML Treatment for Lates calcarifer

XML Treatment for Oreochromis niloticus

XML Treatment for Gambusia affinis

XML Treatment for Xenentodon canciloides

XML Treatment for Pao leiurus

XML Treatment for Parambassis siamensis
